# Job burnout and its influencing factors among village doctors during the COVID-19 pandemic: a cross-sectional study

**DOI:** 10.3389/fpubh.2024.1388831

**Published:** 2024-04-18

**Authors:** Zixuan Zhao, Qiusha Li, Chunxiao Yang, Zhongzheng Zhang, Zhongming Chen, Wenqiang Yin

**Affiliations:** ^1^School of Public Health, Shandong Second Medical University, Weifang, Shandong, China; ^2^Hepatic Biliary Pancreatic Surgery, Yidu Central Hospital of Weifang, Shandong, China; ^3^School of Management, Shandong Second Medical University, Weifang, Shandong, China

**Keywords:** COVID-19 pandemic, village doctors, job burnout, influencing factors, rural health

## Abstract

**Objective:**

The aim of this study is to understand the job burnout of village doctors during the COVID-19 epidemic and its influencing factors, and to provide a reference for effectively alleviating the job burnout of village doctors.

**Methods:**

A cross-sectional survey was conducted among village doctors in S province in December 2021. The survey included a general information questionnaire and the CMBI Burnout Scale. Epidata was used for dual input, and descriptive analysis, *t*-test, chi-square test, and binary Logistic regression for statistical analysis were used.

**Results:**

A total of 993 village doctors participated in the survey. Most of them were male village doctors (62.84%), with an average age of 46.57 (SD = 7.50). Village doctors believed that the impact of the epidemic on work was serious, with a score of 3.87 ± 0.91. The economic support was small, with a score of 2.31 ± 0.99. The development space was low, with a score of 2.62 ± 0.98. The overall incidence of burnout was 53.47%. In the burnout group, 54.05% were mild, 33.14% were moderate, and 12.81% were severe. The high degree of difficulty in using WeChat (*OR* = 1.436, 95%*CI*: 1.229–1.679), high work pressure (*OR* = 1.857, 95%*CI*: 1.409–2.449), high risk of practice (*OR* = 1.138, 95%*CI*: 1.004–1.289), less economic support (*OR* = 0.825, 95%*CI*: 0.684–0.995), less technical support (*OR* = 0.696, 95%*CI*: 0.565–0.858), and poor emotional support (*OR* = 0.632, 95%*CI*: 0.513–0.780) were more likely to have job burnout.

**Conclusion:**

Burnout is a common phenomenon among village doctors during the COVID-19 pandemic, which needs to be prevented and alleviated by various measures.

## 1 Introduction

Attaching importance to the development of grass-roots health services is the goal of China’s medical and health system reform (1). Village doctors in China are responsible for most of the medical care and public health services in rural areas, and are an important part of the medical and health team. Medical care responsibility refers to the basic treatment of common diseases, frequently occurring diseases and chronic diseases, and guides patients to see a doctor in time. Public health service responsibility refers to the establishment of unified and standardized health records for residents in the jurisdiction, regular health knowledge lectures and other 14 national basic public health service projects. Village doctors, as the health “guardians” closest to hundreds of millions of rural residents, have made great contributions to the prevention and treatment of acute and chronic diseases of rural people for many years (2). As a major public health emergency, the epidemic of novel coronavirus pneumonia (Coronavirus disease 2019, COVID-19) has affected a wide range of areas. In addition, sociocultural changes and economic difficulties caused by the prolongation of the epidemic have led to a variety of psychological problems, such as anxiety, depression, panic, burnout, etc. (3–5). Village doctors, as front-line participants in epidemic prevention and control in rural areas, actively fulfilled their duties of screening people from outside and returning home and screening patients with fever. This has played an important role in effectively preventing the spread of the epidemic to rural areas and protecting the health of residents in rural areas (6).

The theory of effort-and-reward imbalance states that the efforts made by individuals at work need to be rewarded in terms of salary, respect, and career development. If there is an imbalance between effort and reward, it will have a negative impact on employee behavior and health, and the long-term imbalance will also lead to negative feelings and tension among staff, leading to job burnout (7). In this epidemic prevention and control, village doctors have done a lot of work, resulting in a sharp increase in their workload, work pressure and practice risks. However, they have not been rewarded in a way that matches their efforts. This may lead to job burnout among village doctors.

Job burnout, also known as burnout or occupational exhaustion, is a psychological syndrome. Job burnout refers to the individual’s long-term stress at work, which leads to physical and mental exhaustion of attitude, emotion, and behavior, resulting in indifference or boredom to work (8, 9). Maslach argues that job burnout is not caused by unilateral causes, either professional or personal, but depends on the balance between personal expectations and professional requirements (10). Job burnout includes three dimensions: emotional exhaustion, depersonalization, and reduced personal accomplishment. The exploration of burnout has deepened in the past few decades. Relevant studies have shown that job burnout exists in various industries (11–13). Among them, the phenomenon of burnout in the medical field is serious, especially in the medical and nursing groups during the COVID-19 epidemic. Scholars such as Chen (14), Vranas (15), Magalh Magalhães (16), and Szczerbińska (17) respectively studied the burnout status and influencing factors of doctors and nurses in large general hospitals during the COVID-19 epidemic. Peng (18), Chen (19), and other scholars studied the current situation and influencing factors of job burnout of medical staff in primary medical institutions during the COVID-19 epidemic. Job burnout of medical staff during the pandemic has become a hot research topic at home and abroad, and it is found that most of the studies are concentrated in tertiary hospitals. In previous studies, there were few investigations on the current situation of job burnout of village doctors since the outbreak of COVID-19. Especially in the regular prevention and control of the COVID-19 epidemic, village doctors are on the front line of managing and maintaining medical services in rural areas. Village doctors are not only facing a higher risk of infection than the general public, but also facing heavy tasks and prominent contradictions between doctors and patients. At the same time, they do not receive the social status, welfare and treatment commensurate with the status of doctors, and more prone to mental health problems. Therefore, it is particularly important to understand the current situation of job burnout of village doctors.

Based on the above background, this study aims to use cross-sectional survey data to analyze the prevalence of job burnout among village doctors in China in the context of COVID-19. And further explore the demographic characteristics, occupational stress, career development support, and other factors on burnout, which is particularly important to effectively alleviate job burnout psychology, so that village doctors maintain a higher combat effectiveness, cohesion and enthusiasm for work.

## 2 Methods

### 2.1 Study design and sampling

S Province is a province in eastern China. By the end of 2021, there are 16 prefecture-level cities (below the provincial level and above the county level) in S province, with a population of 101.527 million, of which 37.513 million are rural residents. The *per capita* disposable income of residents in the province is 35,705 yuan. There are 52,940 village clinics and 97,782 village doctors in S province, with an average of 1.85 village doctors per village clinic (20).

The survey was conducted in December 2021, and the respondents were village doctors. A stratified random sampling method was used to select three cities in S province according to their good, medium, and poor economic levels. In the same way, three counties were randomly selected from each prefecture-level city, three townships were selected from each county, and one village doctor was randomly selected from each village clinic in three townships. The selected personnel went to the township health centers to fill in the questionnaire. Before the investigation, all investigators should receive standardized training. In the survey, a special person is responsible for the quality review of the questionnaire. If problems are found, the respondents will be invited to supplement and improve the questionnaire in time. The questionnaires were reviewed again after the survey, and the unqualified questionnaires were excluded from the follow-up analysis. A total of 1,093 questionnaires were distributed in this research, 100 invalid questionnaires containing missing values were removed using the deletion method and 993 valid questionnaires were recovered, with an effective recovery rate of 90.85% (Effective recovery rate of questionnaires = recovered valid questionnaires/issued questionnaires × 100%). In addition, the survey was conducted anonymously, all participants gave informed consent, and the study protocol was approved by the Ethics Committee of Weifang Medical University (ID: 2021YX-066).

### 2.2 Measurement

#### 2.2.1 General survey

Socio-demographic variables of village doctors, gender (1 = male, 2 = female), age (1 = Under 40, 2 = 40–49 years old, 3 = At least 50 years old), working years (1 = Less than 10 years, 2 = 10–19 years, 3 = 20–29 years, 4 = At least 30 years), educational background (1 = junior middle school or below, 2 = technical secondary school, 3 = junior college or above). Qualification (1 = village doctor license, 2 = licensed assistant doctor, 3 = licensed doctor), difficulty in using WeChat (1 = very small to 5 = very large), annual household income (1 = Less than 30,000 yuan, 2 = 30,000–59,999 yuan, 3 = 60,000–99,999 yuan, 4 = Greater than or equal to 100,000 yuan).

In terms of occupational stress, workload, work pressure, and practice risk are all on the scale of 1–5, 1 = very small, 5 = very large. In the aspect of career development support, economic support, technical support, institutional support, development space, resource support, and emotional support are all on the scale of 1–5, 1 = very unsupportive, 5 = very supportive.

#### 2.2.2 Job burnout

In this study, the CMBI burnout scale revised by Li and Li (21) was used. The scale consists of three dimensions: emotional exhaustion, depersonalization, and reduced personal accomplishment, with a total of 15 items. This scale was developed on the basis of relevant studies at home and abroad, and has been well validated in subsequent large-scale studies (22). At present, the scale is used in China Journal Full-text Database (CJFD). This study changed the seven-point scale to a five-point scale (1 never, 2 rarely, 3 sometimes, 4 often, and 5 always). Because in the pre-survey process, it was found that a large number of respondents said it was difficult to distinguish the answers of the seven-point scale, and the five-point scale was more suitable for Chinese cultural background. Moreover, the five-point classification has been adopted by many scholars (23, 24). In this study, the Cronbach’s alpha coefficients of CMBI and its three dimensions of emotional exhaustion, depersonalization, and reduced sense of achievement were 0.82, 0.93, 0.88, and 0.87, respectively, indicating that the questionnaire had good reliability.

The latest MBI manual (fourth edition) suggests that the scores of the three dimensions should not be accumulated to form a single burnout score (25). Li Yongxin’s criteria (21) were used in this study. In SPSS statistical analysis software, the scores of emotional exhaustion, depersonalization, and reduced sense of achievement were sorted, and the value of 1/3 of each dimension was calculated as the critical value. Among them, emotional exhaustion and depersonalization take the upper tertile, the sense of achievement takes the lower tertile, and burnout occurs when it reaches the standard. Specific critical values are shown in Table 1. We defined zero burnout as the score of the three dimensions did not exceed the critical value. One of the dimensions exceeding the critical value was mild burnout. Two dimensions exceeding the critical value were considered as moderate burnout. Three dimensions exceeding the critical value were severe burnout. The most prominent advantage of this evaluation criterion is that it considers the comprehensive effect of the three factors of job burnout, and the investigation is more comprehensive. In addition, grading the level of burnout will also help to take more targeted prevention and intervention measures.

**Table 1:** Critical value of each dimension of job burnout.

Dimension	Critical value
Emotional exhaustion	>17
Depersonalize	>10
The sense of accomplishment is reduced	<23

Li Yongxin’s criteria were used in this study. The scores of emotional exhaustion, depersonalization, and reduced sense of achievement were sorted, and the value of 1/3 of each dimension was calculated as the critical value. Emotional exhaustion and depersonalization take the upper tertile, the sense of achievement takes the lower tertile, and burnout occurs when it reaches the standard.

### 2.3 Statistical analysis

SPSS 21.0 software was used for statistical analysis. Enumeration data were expressed as frequency and percentage, and measurement data were expressed as x^_^ ± S. The *t*-test and Chi-square test were used to compare the job burnout of different demographic characteristics. Binary Logistic regression analysis was used to identify the influencing factors of job burnout of village doctors. The dependent variable job burnout was treated as a dichotomous variable (zero burnout defined as no burnout occurring, assigned a value of 0, and mild, moderate, and severe burnout defined as burnout occurring, assigned a value of 1). Statistically significant variables in the univariate analysis were included in the regression model as independent variables.

## 3 Results

### 3.1 Socio-demographic characteristics of village doctors

Most of the respondents were male (62.84%), with an average age of 46.57 years (SD = 7.50), and 52.87% were 40–49 years old. 52.87% of village doctors have worked for 20–29 years, and only 2.11% of them have worked for less than 10 years. Among the respondents, 54.38% had secondary school education, 60.32% had village doctor’s license, and 12.19% had licensed doctor’s license. 47.94% of village doctors think that there are some difficulties in using WeChat. The survey found that the annual household income of village doctors was concentrated in 30,000–59,999 yuan (51.06%), and 9.37% of village doctors had an annual household income of more than 100,000 yuan (Table 2).

**Table 2:** Single factor analysis of job burnout of village doctors.

Variables	Total N (%)/x_ ± SD	Burnout N (%)/x_ ± SD	χ^2^/*t*	*p* value
Gender
Male	624 (62.84)	354 (56.73)	7.157	0.007
Female	369 (37.16)	177 (47.97)		
Age
<40	151 (15.21)	80 (52.98)	0.083	0.959
40–49	525 (52.87)	283 (53.90)		
≥ 50	317 (31.92)	168 (53.00)		
Years of service
<10	21 (2.11)	6 (28.57)	6.043	0.110
10–19	168 (16.92)	95 (56.55)		
20–29	525 (52.87)	278 (52.95)		
≥30	279 (28.10)	152 (54.48)		
Academic qualifications
Junior high school and below	9 (0.91)	6 (66.67)	1.208	0.547
Technical secondary school	540 (54.38)	294 (54.44)		
College degree or above	444 (44.71)	231 (52.03)		
Practicing qualification
Village doctor’s license	599 (60.32)	322 (53.76)	1.337	0.512
Assistant practicing physician	273 (27.49)	150 (54.95)		
Medical practitioner	121 (12.19)	59 (48.76)		
Difficulty in using WeChat
Rarely	107 (10.78)	38 (35.51)	48.510	<0.001
Occasionally	143 (14.40)	60 (41.96)		
Sometimes	476 (47.94)	249 (52.31)		
Often	239 (24.07)	162 (67.78)		
Always	28 (2.82)	22 (78.57)		
Annual household income
<30,000	203 (20.44)	117 (57.63)	1.924	0.588
30,000–59,999	507 (51.06)	267 (52.66)		
60,000–99,999	190 (19.13)	100 (52.63)		
≥100,000	93 (9.37)	47 (50.54)		
Workload	3.51 ± 0.61	3.58 ± 0.64	−3.779	<0.001
Work pressure	3.58 ± 0.72	3.78 ± 0.69	−10.031	<0.001
Practice risk	3.18 ± 1.31	3.49 ± 1.26	−8.502	<0.001
Economic support	2.31 ± 0.99	2.04 ± 0.87	9.79	<0.001
Technical support	3.66 ± 0.93	3.40 ± 0.93	10.132	<0.001
Institutional support	3.31 ± 0.89	3.03 ± 0.85	11.450	<0.001
Space for development	2.62 ± 0.98	2.40 ± 0.96	7.857	<0.001
Resource support	3.06 ± 1.07	2.83 ± 1.04	7.262	<0.001
Emotional support	4.00 ± 0.85	3.75 ± 0.87	10.685	<0.001

### 3.2 Occupational stress and support for career development of village doctors

In terms of occupational stress, the scores of workload, work stress and practice risk were 3.51 ± 0.61, 3.58 ± 0.72, and 3.18 ± 1.31, respectively. In the aspect of career development support, the scores of economic support, technical support, institutional support, development space, resource support, and emotional support were 2.31 ± 0.99, 3.66 ± 0.93, 3.31 ± 0.89, 2.62 ± 0.98, 3.06 ± 1.07, and 4.00 ± 0.85, respectively (Table 2).

### 3.3 Job burnout status of village doctors

Of the 993 village doctors interviewed, 46.53% had zero burnout and 53.47% had burnout. In the burnout group, 54.05% were mild, 33.14% were moderate, and 12.81% were severe (Table 3).

**Table 3:** Mild, moderate, and severe job burnout of village doctors.

Degree of burnout	Number of people	Proportion (%)
Mild burnout	287	54.05
Moderate burnout	176	33.14
Severe burnout	68	12.81

Li Yongxin’s criteria were used in this study. One of the dimensions exceeding the critical value was mild burnout. Two dimensions exceeding the critical value were considered as moderate burnout. Three dimensions exceeding the critical value were severe burnout.

### 3.4 Single factor analysis of job burnout of village doctors

The results showed that gender, difficulty in using WeChat, workload, work pressure, practice risk, economic support, technical support, institutional support, development space, resource support, and emotional support had an impact on job burnout of village doctors (*p* < 0.05) (Table 2).

### 3.5 Binary logistic regression analysis on influencing factors of job burnout of village doctors

Burnout was used as the dependent variable (0 = zero burnout, 1 = burnout). Binary Logistic regression analysis was conducted with gender, difficulty in using WeChat, workload, work pressure, practice risk, economic support, technical support, institutional support, development space, resource support, and emotional support as independent variables.

Village doctors with high difficulty in using WeChat are more likely to have job burnout (*p* < 0.001, *OR* = 1.436, 95% *CI*: 1.229–1.679). Job burnout was more likely to occur in respondents with higher work stress (*p* < 0.001, *OR* = 1.857, 95% *CI*: 1.409–2.449). Village doctors with higher practice risk were more likely to have job burnout (*p* < 0.05, *OR* = 1.138, 95% *CI*: 1.004–1.289). The less economic support, the more job burnout of village doctors (*p* < 0.05, *OR* = 0.825, 95% *CI*: 0.684–0.995). Those with less technical support were more likely to have job burnout (*p* < 0.01, *OR* = 0.696, 95% *CI*: 0.565–0.858). The worse the emotional support, the easier the job burnout (*p* < 0.001, *OR* = 0.632, 95% *CI*: 0.513–0.780). Gender, workload, institutional support, development space, and resource support have no effect on job burnout of village doctors (*p* > 0.05) (Table 4).

**Table 4:** Binary logistic regression analysis on influencing factors of job burnout of village doctors.

Variables	β	SE	*p* value	OR	95% CI
Gender	−0.198	0.150	0.187	0.820	0.611,1.101
WeChat is difficult to use	0.362	0.080	<0.001	1.436	1.229,1.679
Workload	−0.172	0.150	0.249	0.842	0.628,1.128
Work pressure	0.619	0.141	<0.001	1.857	1.409,2.449
Practice risk	0.129	0.064	0.043	1.138	1.004,1.289
Economic support	−0.192	0.095	0.044	0.825	0.684,0.995
Technical support	−0.362	0.107	0.001	0.696	0.565,0.858
Institutional support	−0.149	0.125	0.233	0.861	0.674,1.101
Development space	−0.082	0.087	0.343	0.921	0.777,1.092
Resource support	0.030	0.086	0.730	1.030	0.871,1.219
Emotional support	−0.458	0.107	<0.001	0.632	0.513,0.780

## 4 Discussion

In this study, 993 village doctors in S Province were surveyed and interviewed to explore the current situation and influencing factors of job burnout of village doctors in China during the COVID-19 epidemic. The results showed that the job burnout rate of village doctors was 53.47%. The degree of difficulty in using WeChat, work pressure, practice risk, economic support, technical support, and emotional support all have an impact on the job burnout of village doctors during the epidemic.

The study found that the burnout rate of village doctors in China during the COVID-19 pandemic was 53.47%. Chinese scholar Zhao et al. (26) investigated the burnout of village doctors during the epidemic prevention and control period, and the detection rate of burnout was 56.3%. Chen Yunfeng (19), Chen Dongran (27), and other scholars surveyed grass-roots medical staff in Shanghai and Xinjiang during the COVID-19 epidemic, and found that the burnout rates were 63.6 and 48.5%, respectively. The results of the above studies are similar to those of this study. According to the research of Hain et al. (28), a South African scholar at the same time, 68.5% of village doctors suffered from burnout during the COVID-19 epidemic. Austrian scholar Kurzthaler et al. (29) and Australian scholar Hoffman et al. (30) assessed the prevalence of burnout among general practitioners during the COVID-19 pandemic, which was 70.0 and 75.0%, respectively. These studies were higher than the detection rate of job burnout of village doctors in this study.

Village doctors who have difficulty in using WeChat have a higher rate of job burnout, which is consistent with the research of Li Xungui and Parkinson (31, 32). The reason may be that the unbalanced age structure of village doctors was found during the survey, with a relatively high proportion of doctors aged over 50. The older people are, the less able they are to accept new knowledge and technology, which leads to the difficulty of using network tools for village doctors. However, during the COVID-19 pandemic, the work of village doctors is highly dependent on communication tools, which is a burden for village doctors with heavy tasks and older age. Job burnout can easily result from job demands that exceed one’s ability (28).

Village doctors with greater work pressure are more likely to suffer from job burnout, which is consistent with the research results of scholars such as La and Yuan Beibei (33, 34). The reason may be that village doctors, as grass-roots front-line personnel in the fight against the epidemic, not only need to provide basic medical and public health services, but also need to undertake temperature monitoring, nucleic acid collection, health education and other work. Epidemic prevention and control work not only increased the working hours of village doctors, but also increased the intensity of work. Studies have shown that in the state of overload, doctors not only cannot provide more effective services for patients, but also are harmful to physical and mental health, and are prone to job burnout (35).

Village doctors with higher practice risk have a higher level of job burnout, which is consistent with the research of scholars such as Jia Haiyi and Izhar (36, 37). Analysis of the possible reasons, first, the novel coronavirus pneumonia has the characteristics of strong infectivity and fast transmission. Village doctors, as front-line anti-epidemic personnel, are facing a greater risk of infection, and it is very easy to have burnout in their work if their own safety is not guaranteed. Second, the closure of the epidemic has brought tremendous psychological pressure to the public, and problems such as anxiety and depression occur frequently. This also invisibly increases the difficulty and risk of the work of village doctors. At present, the risk-sharing mechanism of village doctors is not sound, and village doctors in most areas need to take full responsibility in the face of medical risks. The current situation of “high risk, low security” makes village doctors avoid their work, affects their work enthusiasm, and then produces burnout psychology.

Village doctors with less economic support are more likely to suffer from job burnout. Analysis of the reasons may be, first, village doctors affected by the epidemic, increased workload, extended working hours, but the source of income is still composed of general medical fees, public health subsidies, and basic drug subsidies. The workload of the epidemic surge has not been matched by the income, and the imbalance between pay and return can easily lead to burnout. Second, at present, village doctors cannot enjoy the pension insurance benefits of enterprise employees, but can only participate in the medical insurance of urban and rural residents as residents, and the level of pension security is poor. However, ERG theory mentions that old-age security is a basic survival need, and unmet survival needs are important factors affecting job satisfaction, career choice, turnover intention, and job burnout (38–40).

Village doctors with less technical support have a higher rate of job burnout, which is consistent with the findings of Cohen et al. (41). First, on-the-job training is the main way for village doctors to receive external technical support, but the task of village doctors is arduous. After the village doctors participated in the training, no one took over the work, resulting in insufficient training time. Village doctors have no time to learn and make progress, and their development space is limited, which aggravates their burnout. Second, during the epidemic period, due to the impact of control, village doctors participated in training mostly in township hospitals, and the training platform limited the opportunities for village doctors to contact higher-level doctors. This situation is not conducive to the sustainable development of village doctors themselves, nor to their future career planning, which leads to burnout. Thirdly, the participation of village doctors in training is mainly based on distance/video teaching, which is out of touch with practice. This training mode is not conducive to the improvement of their own technical level, and the career development needs of village doctors cannot be met. In addition, the training content is mostly focused on epidemic prevention and control, and there is a phenomenon of inadequate training. This makes it more difficult for village doctors to participate in epidemic prevention and control work, and they are prone to make mistakes, which aggravates burnout (42).

The worse the emotional support, the higher the level of job burnout of village doctors. First, the support of superior leaders can stimulate the work motivation of village doctors and fully tap their work potential. On the contrary, the weakening of leadership emotional support will reduce the enthusiasm of village doctors, and then lead to burnout. Second, village doctors can devote themselves more wholeheartedly to their work with the support of their families. During the epidemic, village doctors faced a greater risk of infection, isolation, lack of family and friends, and the weakening of emotional support exacerbated the emotional exhaustion of village doctors (43).

The advantage of this study is that there have been few reports on the job burnout of village doctors since the outbreak of COVID-19. This study explored the current situation of job burnout among village doctors under the specific situation of the novel coronavirus epidemic, and further identified its influencing factors. On the one hand, it enriches the relevant data on job burnout at the grassroots level and provides new evidence and perspective for the study of job burnout. On the other hand, more targeted suggestions can be made to improve the phenomenon of job burnout, which will help the stability and development of the village doctor workforce and is important for the promotion of primary health care. However, the following limitations of this study have to be recognized. First of all, the questionnaire data in this study were obtained through the respondents’ self-filling, and there would inevitably be recall bias, which would have a certain impact on the results. Second, in cross-sectional study design, causality is difficult to determine, the future needs to conduct longitudinal research. Finally, although this study has explored a series of influences on job burnout, it has yet to evaluate the protective factors of job burnout such as psychotherapy, counseling interventions, etc. These limitations should be considered and further improved in the follow-up study.

## 5 Conclusion

Since the new medical reform, the government has issued a series of policies to promote the development of village doctors, but village doctors are still a weak link in the medical and health system. This study investigates the current situation and influencing factors of job burnout of village doctors in China under the background of COVID-19, which provides a reference for more targeted elimination of burnout psychology of village doctors. The results show that the burnout rate of village doctors is generally high. Job burnout is related to factors such as difficulty in using WeChat, high work pressure, high practice risk, less economic support, less technical support, and poor emotional support. In order to effectively alleviate the job burnout of village doctors, it is necessary to ensure that government subsidies are in place in full and on time, especially to ensure that the income of village doctors is commensurate with their efforts during the epidemic. In addition, we should design and improve the old-age security mechanism of village doctors, improve the old-age security treatment, and effectively alleviate the job burnout of village doctors.

## Data availability statement

The original contributions presented in the study are included in the article, further inquiries can be directed to the corresponding authors.

## Ethics statement

This study was approved by the Ethics Committee of Weifang Medical University. Participants provided consent to participate in the study.

## Author contributions

ZiZ: Data curation, Methodology, Writing – original draft, Writing – review & editing. QL: Methodology, Supervision, Writing – original draft, Writing – review & editing. CY: Data curation, Supervision, Writing – original draft, Writing – review & editing. ZhZ: Data curation, Supervision, Writing – original draft, Writing – review & editing. ZC: Funding acquisition, Supervision, Writing – original draft, Writing – review & editing. WY Funding acquisition, Supervision, Writing – original draft, Writing – review & editing.
